# Ten Minutes in Medical Electricity

**Published:** 1895-07

**Authors:** Geo. S. Hull

**Affiliations:** Chambersburg, Pa.


### TEN MINUTES IN MEDICAL ELECTRICITY.

BY GEO. S. HULL, PH. G., M. D., CHAMBERSBURG, PA.

I have thought best to spend the ten minutes allotted to this paper, in speaking, first, of some of the sure effects of electricity, and then of some phases of electric humbuggery.

Under the electrolytic effects upon which we can always depend, I will consider, first and tersely, the removal of superfluous hairs by the galvanic current. Eight or ten Leclanche cells will furnish us the desired current, when connected in series. We select this well-known cell, because it is inexpensive, free from odor, cleanly, and easily kept in order. Physicians not able to purchase the "regular outfit" can readily set up this simple battery. The handle carrying the needle is connected by means of a conducting cord of ample thickness with the zinc rod, making it the negative electrode. The cord leading from the carbon, in the porus cup, is attached to a metal or carbon disc, covered with chamois skin, and is the positive electrode. When the needle is inserted along the hair and to its root, the current is made by pressing the moistened positive electrode against the palm of the hand, or by dipping the fingers into a bowl of water in which the electrode is immersed. Soon a few bubbles of hydrogen escape along the needle, and shortly very gentle traction will remove the hair.

A brief practice will enable one to ascertain just how long to keep the needle in so as to effctually destroy the hair follicle. Too short a time, especially with a feeble current, means failure; too long a time, or too strong a current, may mean a scar more or less serious. Hairs which return, should receive a second application of the current, which is fatal to them. Sometimes they are so much diseased that a simple extraction of them is sufficient. It is not well to remove too many hairs in close proximity, as the coalescing of several trifling points of inflammatory action may result in a deforming abscess.

The needle which is preferred by the writer, is of steel, and is neither thick, stiff nor sharp. If a needle can be made flexible enough to follow a hair to its root, when its course is not in the direction it seems to be, then the ideal needle is at hand. A good light, a strong lens (10 to 12 dioptres), and a steady hand, are necessary requisites. It is well to limit one's self to from forty to sixty hairs at one sitting, to do it well; and, if carefully done, the percentage of returns will be small, not over five or ten per cent. Complaints of pain are but seldom made during the operation, and the reaction from the punctures, which is like that from the bite of a mosquito, passes away in a few hours.

When the electrolytic action seems to be lessening, as evidenced by the tardiness with which the bubbles of gas make their appearance at the needle, two things need be looked after: first, that the connections with the cells are all clean and tight; second, that the liquid in the cells is up to the required height (within one and a half or two inches of the top of each cell). If still the action is deficient, some ammonium chloride should be put in each cell (originally the cells had six ounces in each). If still no betterment, and the zinc rods are in fair condition, then the depolarizing material in the porous cups surrounding the carbon is exhausted, and cups like the ones removed must be purchased. Sometimes if the cells are used for too long a time at a sitting, or sittings are repeated at very short intervals, the current runs down from polarization and all that is needed is to rest the battery for a short time. One should limit the number of cells used to the effect, not aiming to "burn out" the hairs too rapidly; allowing from ten to twenty seconds for each hair.

Once in awhile, we hear of the current from an induction coil being tried by one who thinks that electricity is the same in its effects, no matter from what source it comes; he generally succeeds in producing plenty of pain and mental impression, but the hair remains intact. What is demanded is a continuous current of sufficient electro-motive force to furnish the required strength of current working through the high resistance of the body. The faradic current, from the induction coil in the ordinary physician's battery, gives plenty of electro-motive force, but it is constantly reversing its direction and gives far too little quantity.

After becoming expert in removing hairs, one soon learns to remove moles, warts and other blemishes.

The so-called galvanic current from a battery of from 25 to 100 cells, is used, though without such uniform success, in many uterine disorders. In such use, however, both the galvanic and faradic currents must be thoroughly understood to be rightly and even safely applied. Only the skillful should be trusted here, where milliamperemeters and rheostats and carefully selected induced currents are necessities. Abnormal growths may be resolved by the use of the galvanic current, while normal tissues may be built up by the faradic. There is a vast difference, however, between the appliances and methods used when one removes a malignant growth by bipolar electrolysis and then applies the zinc-amalgam cataphoresis to the cavity, and when one simply applies the faradic current to a relaxed organ.

This large and tempting field, our ten minute's limit compels us to pass over with merely the mention of it.

We must say a word about the batteries used for electro-cautery, which is merely the use of a heated loop or spiral of wire which can be placed in position cold and almost instantly heated to any desired temperature. Of course, the faradic current from our ordinary "physician's battery" will not do for this; and our Leclanche cells, in series, as described, will not give sufficient quantity to heat the thickness of wire generally used. The cells should have large zinc and carbon plates, so as to get abundance of chemical action. Two or three such cells, in series, will generally prove sufficient. We want a large quantity of current under low electro-motive force, as the resistance of the circuit is small.

A good storage battery is both convenient and safe. It may be charged from the street mains at night, and used during the day, as desired; or the current direct from such mains may, with proper regulation, be used without danger. The same cannot be said of such current when used for electrolysis, even with the best regulators.

It must be admitted that electricity, properly applied, is a most valuable therapeutic agent, but it is far from a cure-all. The extraordinary claims of some electropaths are unwarranted, and the bold assertions of advertisers, who flood the medical market with multiform electric devices, are often ridiculous in the extreme. But electric humbuggery seems to have fastened itself upon the people; they like it and pay well for it. One man wears an "electric belt" for his weak back, which can only strengthen the weak muscles there, by its added weight of zinc and copper giving them some additional exercise, or its irritation stimulating the circulation of the parts, or his faith in its curing some imaginary trouble in the parts. There is no electricity worth speaking of generated by the device, and if so, no way of getting it to the muscles. Another man "smells the electricity" from an "electric inhaler" after having been "out with the boys," and the pungent odor of the oil of mustard does what it can to revivify him. It is claimed for this same "inhaler" that it will pass so much electricity through the skin in cases of neuralgia that it will blister the surface—it really does, but a mustard plaster, not so handsome as the plated "inhaler," is identical with it in every other respect, and there is no more electricity in the "inhaler" than in the mustard plaster. An elderly lady wears an "electric headband with an extra attachment for removing wrinkles" by increasing the tissues of the parts, while a younger dame puts on an "electric corset" which will remove excessive stoutness, and "has been worn by Lady Jersey and other ladies." These devices, with such inconsistent and ridiculous claims, were actually on exhibition at our recent Columbian Exhibition. A prematurely aged man uses an "electric hairbrush" on his bald pate, which, if the bristles are stiff, may help him by friction, but not by virtue of any electricity in it. The brush may seem to establish its claims by attracting the needle of a small compass, but the feeble ampelrian currents in the concealed magnet never get to the hair follicles. Then there are "electric towels," which actually have induction coils attached to them, and one can acutely feel the current; but, instead of being satisfied in pushing these solely on their merits as being good for stimulating the skin (and hard to keep in order), they claim for them the power of destroying typhoid fever germs, and all other pathogenic germs for which they can find names. These and many other so-called wonderful achievements of electricity are not new, though still startling. But now, we have to record the surprising claim that electricity, properly applied, has to do with getting oxygen into the blood through the skin. As "devitalization of the blood" is claimed to be the "sole cause of disease," therefore an instrument (which nobody understands but the inventor) to produce "polar attraction" on the surface of the body, so the oxygen can be absorbed, is all that is required. "All poisonous medicines are strictly forbidden" or the "polarizer" will not work. As the materials in the "polarizer" are "imperishable," and the "value of each instrument increases with age," fifty dollars is, of course, a very moderate price to pay for one.

But the very claim that the material which generates the force is imperishable, proves at once, and conclusively, that there is no electricity about the device, and, granting that electricity was capable of driving oxygen into the blood vessels, the question might be asked, with propriety, whether the lungs should be thus interfered with in their function. Right here, I would modestly venture a suggestion that it is not oxygen that the "polarizer" causes the skin to absorb, but the new element in the air, argon. So little is yet known about argon that the claim that it produces miraculous effects upon the system, when driven in through the skin, might be hard to disprove. So, if the Ralstonians, whose "glame" in the air might be identical with argon, do not claim priority, why should not the "polarizer" people patent the argon idea.

To lie down upon a downy couch when one is overworked or ill, and by a conducting cord running from one's ankle to a "polarizer," and another cord leading from the instrument with its "imperishable" material to the moist earth, or a cake of ice:—to lie down and even cease from the labor of breathing, and have this perpetual-source-of-energy machine force oxygen, or argon, or something even more mysterious into one's blood and give new life to every part—'twere a "consummation devoutly to be wished."

